# Indolent T-cell lymphoproliferative disorder of the gastrointestinal tract: a tricky diagnosis of a gastric case

**DOI:** 10.1186/s12876-020-01488-5

**Published:** 2020-10-14

**Authors:** Magda Zanelli, Maurizio Zizzo, Francesca Sanguedolce, Giovanni Martino, Alessandra Soriano, Stefano Ricci, Carolina Castro Ruiz, Valerio Annessi, Stefano Ascani

**Affiliations:** 1 Pathology Unit, Azienda Unità Sanitaria Locale-IRCCS di Reggio Emilia, Reggio Emilia, Italy; 2 Surgical Oncology Unit, Azienda Unità Sanitaria Locale-IRCCS di Reggio Emilia, Reggio Emilia, Italy; 3 Clinical and Experimental Medicine PhD Program, University of Modena and Reggio Emilia, Modena, Italy; 4 Pathology Unit, Azienda Ospedaliera Universitaria “Ospedali Riuniti” di Foggia, Foggia, Italy; 5 Hematology Unit, CREO, Azienda Ospedaliera Di Perugia, University of Perugia, Perugia, Italy; 6 Gastroenterology Unit, Azienda Unità Sanitaria Locale-IRCCS di Reggio Emilia, Reggio Emilia, Italy; 7 Pathology Unit, Ospedale Di Terni, University of Perugia, Perugia, Italy

**Keywords:** Stomach, Gastrointestinal tract, Indolent, T-cell, Lymphoproliferative disorder

## Abstract

**Background:**

Indolent T-cell lymphoproliferative disorder of the gastrointestinal tract is a rare low-grade clonal lymphoid proliferation, included as a provisional entity in the current World Health Organization classification. The disease is generally localized to the gastrointestinal tract, mainly small bowel and colon. Involvement of other organs is infrequently reported. The majority of patients show a protracted clinical course with persistent disease. A prolonged survival, even without treatment, is common.

**Case presentation:**

A 28-year-old woman had a 2-year history of dyspepsia and lactose intolerance. Autoimmune diseases and celiac disease were excluded. No gross lesions were identified by endoscopy. Multiple gastric biopsies showed a small-sized lymphoid infiltrate, expanding the lamina propria, with a non-destructive appearance. The lymphoid cells were positive for CD3, CD4, CD5, CD7 and negative for CD20, CD8, CD56, CD103, PD1, CD30, ALK1, CD10, BCL6, perforin, TIA-1, Granzyme B and Epstein-Barr virus-encoded RNA. KI-67 index was low (5%). Molecular analysis revealed a clonal T-cell receptor γ rearrangement. Bone marrow was microscopically free of disease, but molecular testing identified the same T-cell receptor γ rearrangement present in the gastric biopsies. After the diagnosis of indolent T-cell lymphoproliferative disorder of the gastrointestinal tract, the patient received steroid therapy, only for 2 months. She is alive, with a stable disease restricted to the stomach, at 12 months from diagnosis.

**Conclusions:**

Indolent T-cell lymphoproliferative disorder is usually a disease of adulthood (median age: 51 yrs). The small bowel and colon are the sites most commonly involved. Our case occurred in a young woman and affected the stomach, sparing small intestine and colon. Clonality testing identified involvement of bone marrow, a site infrequently affected in this disease. Our aim is focusing on the main diagnostic issues. If appropriate immunostainings and molecular analysis are not performed, the subtle infiltrate may be easily overlooked. The risk of misdiagnosis as more aggressive lymphomas, causing patient overtreatment, needs also to be considered.

## Background

The gastrointestinal (GI) tract is the most common extra-nodal site of involvement for non-Hodgkin lymphomas [1]. T-cell lymphomas primarily or secondarily involving the GI tract are aggressive diseases in the vast majority of cases [1]. Indolent T-cell lymphoproliferative disorder (ITLPD) is a non-aggressive, clonal, mature T-cell disorder, which can affect almost any part of the GI tract, although it is more uncommon in stomach, esophagus and oral cavity [1–7]. Patients often show prolonged survival even without any treatment [1–7]. In daily routine, GI biopsies evaluation represents a common challenge for pathologists, who have to make timely and accurate diagnoses, in order to establish the adequate treatment.

We report a case of ITLPD in a young female with mild gastric symptoms. The disease was limited to the stomach, without small bowel and colon involvement. Clonal T-cell receptor γ rearrangement was identified in the bone marrow. The disease remained stable without chemotherapy, at 12 months from diagnosis. We focus on the main potential diagnostic pitfalls, which can cause inappropriate patient management.

## Case presentation

**Fig. 1:**
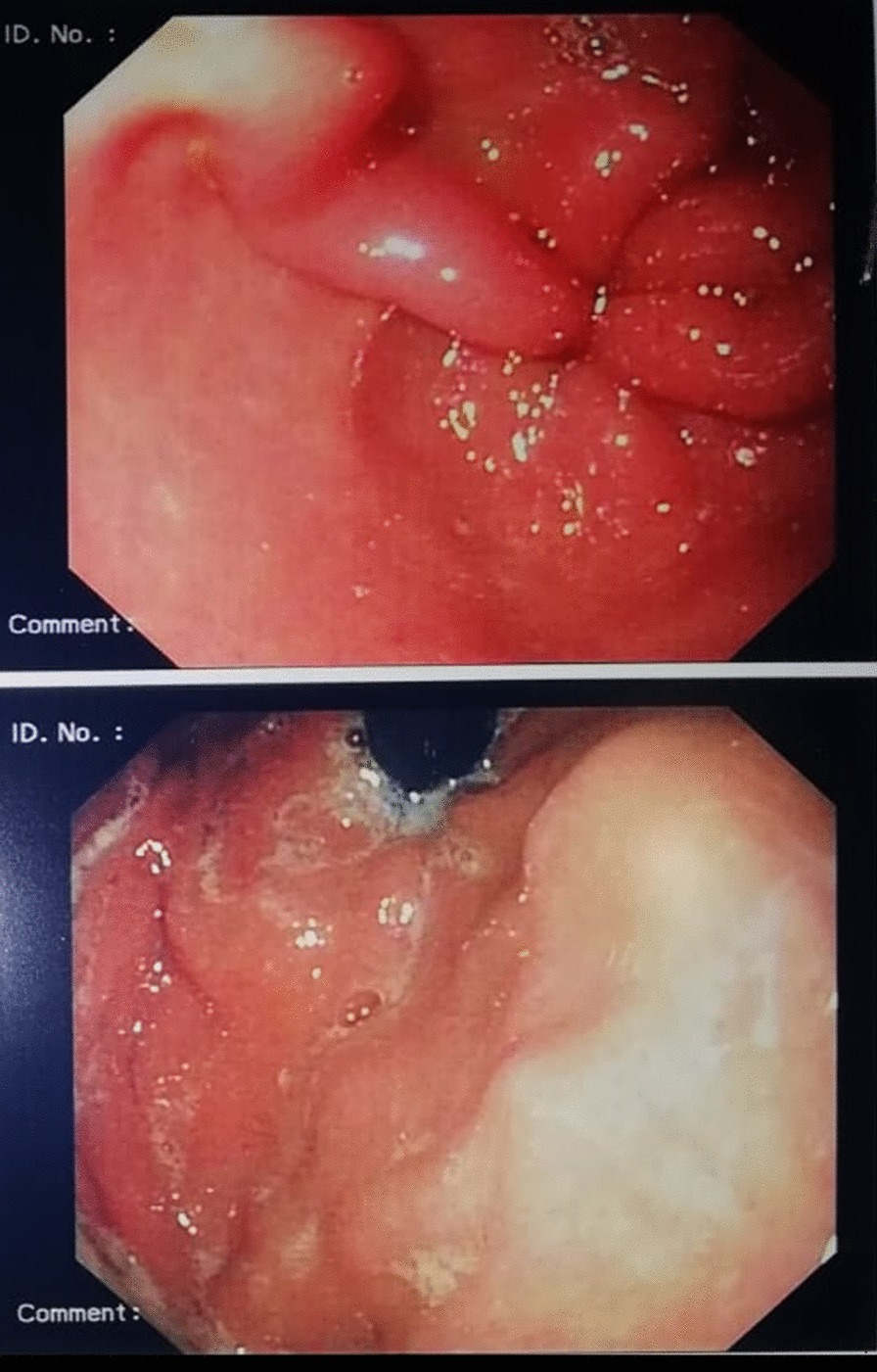
Endoscopic view showing hyperemic gastric mucosa

A 28-year-old immunocompetent woman presented with a 2-year history of lactose intolerance and dyspepsia which had been worsening in the last 6 months. Blood exams and lactate dehydrogenase (LDH) level were within normal limits. Laboratory tests ruled out celiac disease as well as other autoimmune diseases. No hepatosplenomegaly and lymphadenopathy were present. By upper gastrointestinal endoscopy, the esophagus showed normal caliber and morphology and the overlying mucosa was unremarkable. A mild incontinence of the cardia was observed. The stomach was normally distensible and mainly the gastric body mucosa appeared mildly hyperemic and edematous (Fig. 1). The pylorus was symmetric; the bulb as well as the second duodenal portion were unremarkable.

**Fig. 2:**
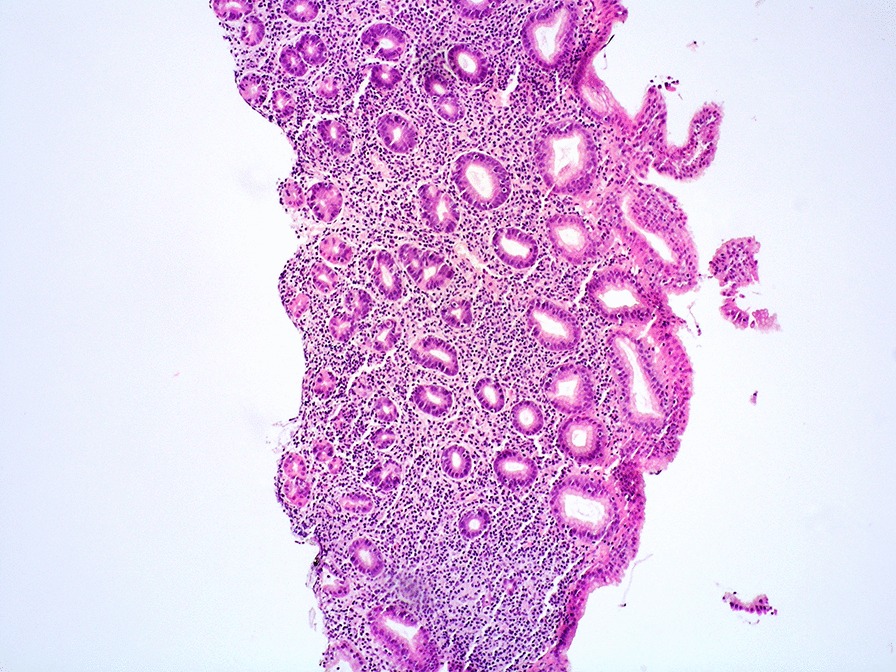
Medium power view showing lymphocytes expanding the lamina propria of gastric mucosa. The glands are preserved with few lymphocytes invading the epithelium (HE 200 × magnification)

**Fig. 3:**
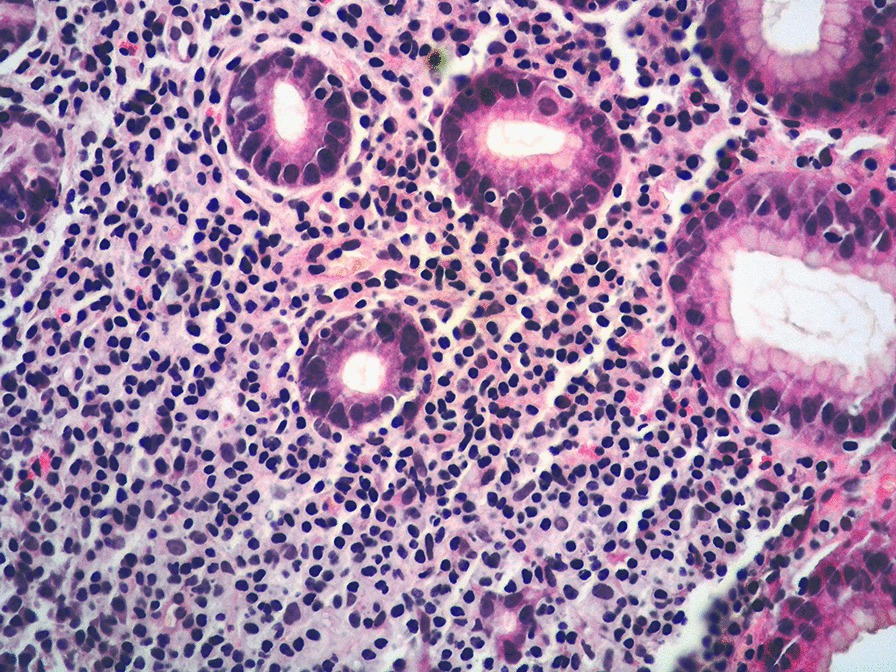
High power view highlighting cytological details of the infiltrate made up of small-sized, mature-appearing lymphocytes (HE 400 × magnification)

**Fig. 4:**
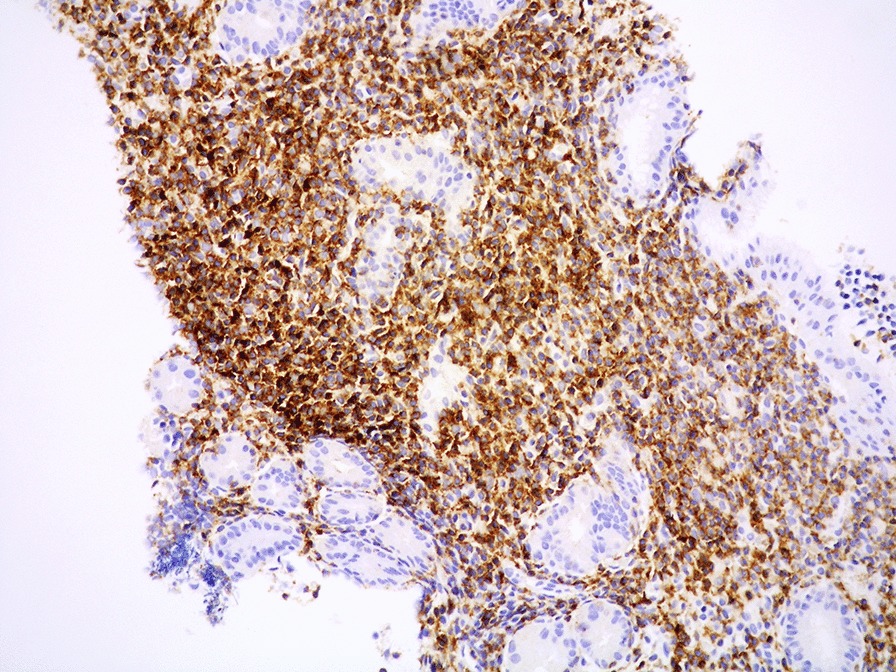
Mucosal lymphoid infiltrate diffusely CD4-positive (immunostain)

Microscopically, the lamina propria showed a subtle, non-destructive and monotonous lymphoid infiltrate composed of small lymphocytes (Figs. 2, 3), focally invading the glandular epithelium and realizing rare lymphoepithelial lesions (LELs). Few eosinophils and plasma cells were admixed. The lymphoid cells were diffusely positive for T-cell markers such as CD3, CD4 (Fig. 4), CD5 and CD7 and negative for CD20, CD8, CD56, CD103, PD1, CD30, ALK1, CD10, BCL6, perforin, TIA-1 and Granzyme B. In situ hybridization for Epstein-Barr virus (EBV)-encoded RNA (EBER) was negative. The Ki67 proliferative fraction was low (5%). PCR-based T-cell receptor (TCR) clonality testing was performed [8] and, according to EuroClonality/BIOMED-2 guidelines, clonal T-cell receptor γ (TCRγ) rearrangement was reported. The case was interpreted as ITLPD. Bone marrow biopsy showed a subtle and mild, interstitial lymphoid infiltrate with a mixed immunohistochemical phenotype. However, molecular testing identified an identical TCRγ rearrangement. Colonoscopy was negative. Multiple biopsies from small intestine and colon were taken. Immunohistochemistry and molecular analysis ruled out the presence of disease in small and large bowel. Computerized tomography (CT) scan and positron emission tomography (PET) scan were negative. Prednisone (50 mg daily) was administered for 2 months. GI biopsies performed 12 months after the initial diagnosis, confirmed a stable and persistent disease limited to the gastric mucosa. Due to lack of symptoms, the steroid therapy was stopped and a wait and see policy adopted.

## Discussion and conclusions

ITLPD of the GI tract is a rare low-grade clonal lymphoid proliferation, included as a provisional entity in the current World Health Organization (WHO) classification [1]. The etiology is unknown, although few cases are reported in patients with inflammatory bowel disease and autoimmune disorders [2–7]. Any part of the GI tract can be involved with single or multiple lesions [2–7]. The small intestine and colon are the sites most frequently affected. Involvement of the stomach, as occurred in our case, is uncommon as well as esophageal and oral involvement [7]. ITLPD can show heterogeneous phenotype: most cases express CD8, some express CD4 and rare are double negative [1]. The identification of clonal rearrangement of TCR (αβ or γ) is essential for the diagnosis [1]. ITLPD remains localized to the GI tract often for a long duration, with chronic relapsing clinical course. Prolonged survival with persistent disease is common; conventional chemotherapy is usually not effective [1, 7]. Dissemination to other organs can be observed with disease progression [1, 4]. Bone marrow involvement has been infrequently reported [5, 9, 10]. Higher-grade T-cell lymphomas may rarely develop in a subset of cases [1].

Our case underlines how challenging can be the recognition of this entity for both clinicians and pathologists. ITLPD of the GI tract can be either overlooked or misdiagnosed as an aggressive lymphoma. Firstly, the symptoms are non-specific (abdominal pain, diarrhea, vomiting, food intolerance, dyspepsia, bleeding) and commonly lasting for long time before diagnosis. ITLPD is often clinically misinterpreted as refractory celiac disease or inflammatory bowel disease (IBD) with delay in diagnosis [1–7]. Secondly, the endoscopic features are non-specific as well. The mucosa can appear normal or slightly hyperemic, as in our case. Sometimes prominent folds, erosions or nodules are present [2–7]. Lastly, the mucosal lymphoid infiltrate is subtle, generally limited to the mucosa and rarely extending to the submucosa, without forming tumor masses [2–7]. The infiltrate can be easily overlooked, if appropriate immunohistochemical and molecular analyses are not performed. A misleading aberrant expression of B-cell markers, such as CD20, has been reported in one case of ITLPD by Wang et al. [9]. It needs to be emphasized that ITLPD diagnosis always requires clonality testing. All cases of ITLPD show clonal rearrangement of TCR genes, either TCRβ or TCRγ [1–5]. Recent data suggest that ITLPD are genetically heterogeneous and share some pathogenetic mechanisms with other intestinal T-cell lymphomas as mutations in the JAK-STAT signaling pathway [11]. Genetic abnormalities involving TET2, DNMT3A, KMT2D genes have also been recently reported [11].

It is mandatory to distinguish ITLPD from aggressive T-cell lymphomas, which can involve the GI tract, in order to avoid unnecessary therapy. In aggressive lymphomas, such as monomorphic epitheliotropic intestinal T-cell lymphoma (MEITL) and enteropathy associated T-cell lymphoma (EATL), about half of the patients present acutely with intestinal obstruction or perforation, although others have an insidious presentation with abdominal pain and diarrhea, more similar to ITLPD [1, 6, 7]. In EATL and MEITL, the lymphoid infiltrate is generally transmural, with a destructive pattern of growth. Pleomorphism is more common in EATL, whereas in MEITL the cells have a more uniform appearance. The proliferation index is high [1, 6, 7]. Both EATL and MEITL are aggressive neoplasms not responding to current therapy. Differently, in ITLPD the infiltrate is often limited to the mucosa, without forming a mass, it is bland-looking with a low proliferative fraction. Extranodal, NK/T-cell lymphoma, nasal-type, needs to be ruled out. It arises mostly in the upper aerodigestive tract. GI involvement can occur generally later in the course of the disease or more rarely the GI tract represents the primary site of the disease [1, 5]. Necrosis, angiocentricity, angioinvasion and EBER positivity are characteristics features of this aggressive lymphoma, typically absent in ITLPD. To be mentioned the indolent NK-cell proliferation of the GI tract, variably labelled as NK-cell enteropathy and lymphomatoid gastropathy [7]. The clinical course is often indolent and protracted, like in ITLPD. EBER negativity is helpful to rule out extranodal-NK/T-cell lymphoma, nasal type, which represents the major diagnostic pitfall. Other entities, typically presenting outside the GI tract, as peripheral T-cell lymphoma not otherwise specified or anaplastic large cell lymphoma may sometimes present as intestinal disease [1]. Clinico-pathological correlation is essential to reach the correct diagnosis.

In conclusion, we report a case of CD4-positive ITLPD with gastric presentation, an uncommon site compared to small and large bowel. In absence of chemotherapy, the disease remained confined to the stomach, at 12 months from the initial biopsy. Despite bone marrow appeared disease-free histologically, T-cell clonal rearrangement was identified in bone marrow. ITLPD usually remains localized to the GI tract for years and involvement of other sites such as bone marrow is rare and usually observed with disease progression. Clinicians and pathologists need to be aware of ITLPD as, in order to make the correct diagnosis, clinico-pathological correlation is essential. After having ruled out autoimmune disorders, a crucial point in the diagnosis of ITLPD is the identification of a bland T-cell infiltrate, usually limited to the mucosa, resulting monoclonal with molecular analysis.

## Data Availability

All the original data supporting our research are described in the Case presentation section and in the figures' legends.

## Abbreviations

GI: Gastrointestinal
ITLPD: Indolent T-cell lymphoproliferative disorder
LDH: Lactate dehydrogenase
LEL: Lymphoepithelial lesion
EBV: Epstein-Barr virus
EBER: EBV-encoded RNA
WHO: World Health Organization
IBD: Inflammatory bowel disease
MEITL: Monomorphic epitheliotropic intestinal T-cell lymphoma
EATL: Enteropathy associated T-cell lymphoma
